# Supplementary material for: Theory of the Kitaev model in a [111] magnetic field
Source: Nat Commun. 2022 Jan 20;13:399. doi: 10.1038/s41467-022-28014-3 (PMC8776843; doi:10.1038/s41467-022-28014-3)
Supplement: Supplementary file 1 — Supplementary Information [file 41467_2022_28014_MOESM1_ESM.pdf]

Supplementary Information for "Theory of the Kitaev model in a [111] magnetic field"

I. DIMENSIONLESS PARAMETERS OF THE EFFECTIVE HAMILTONIAN

Here we derive the momentum-space hybridization parameters, $P_{\mathbf{k},\alpha} = \sum_{\mathbf{R}} p_{\mathbf{R},\alpha}\, e^{i\mathbf{k}\cdot\mathbf{R}}$, and the flux-pair hopping parameter q in Eq. (6) of the main text. We first consider the hybridization parameters. Using the momentum-space complex matter fermions diagonalizing the flux-free sector of the pure Kitaev model,

$$\psi_{\mathbf{k}} = \frac{1}{\sqrt{2}}\left(C_{\mathbf{k},A} + i e^{i\varphi_{\mathbf{k}}} C_{\mathbf{k},B}\right) = \frac{1}{2\sqrt{N}} \sum_{\mathbf{r}\in A} c_{\mathbf{r}} e^{-i\mathbf{k}\cdot\mathbf{r}} + \frac{i}{2\sqrt{N}} \sum_{\mathbf{r}\in B} c_{\mathbf{r}} e^{-i\mathbf{k}\cdot\mathbf{r}+i\varphi_{\mathbf{k}}}, \tag{S1}$$

where $e^{i\varphi_{\mathbf{k}}} = \lambda_{\mathbf{k}}/|\lambda_{\mathbf{k}}|$ and $\lambda_{\mathbf{k}} = \sum_{\alpha} e^{i\mathbf{k}\cdot\hat{\mathbf{r}}_\alpha}$, the hybridization term in Eq. (4) of the main text can then be written as

$$\begin{aligned}\tilde{\mathcal{H}}_p = &\frac{h}{\sqrt{N}} \sum_{\alpha} \sum_{\mathbf{k}} \sum_{\mathbf{r}\in A} \left[i(\tilde{\chi}_{\mathbf{r}}^{\alpha})^\dagger \psi_{\mathbf{k}} e^{i\mathbf{k}\cdot\mathbf{r}} \left(P_{\mathbf{k},\alpha} + P_{-\mathbf{k},\alpha} e^{i\mathbf{k}\cdot\hat{\mathbf{r}}_\alpha - i\varphi_{\mathbf{k}}} \right) + \text{H.c.} \right] \\ &+ \frac{h}{\sqrt{N}} \sum_{\alpha} \sum_{\mathbf{k}} \sum_{\mathbf{r}\in A} \left[i(\tilde{\chi}_{\mathbf{r}}^{\alpha})^\dagger \psi_{-\mathbf{k}}^\dagger e^{i\mathbf{k}\cdot\mathbf{r}} \left(P_{\mathbf{k},\alpha} - P_{-\mathbf{k},\alpha} e^{i\mathbf{k}\cdot\hat{\mathbf{r}}_\alpha - i\varphi_{\mathbf{k}}} \right) + \text{H.c.} \right].\end{aligned} \tag{S2}$$

The two terms in Eq. (S2) can be matched with the microscopic model by considering the matrix elements of the bare Zeeman term, $\mathcal{H}_h = h \sum_\alpha \sum_{\mathbf{r}} i b_{\mathbf{r}}^\alpha c_{\mathbf{r}}$, between appropriate states of the pure Kitaev model: the ground state, $|\Omega\rangle = |\omega\rangle \otimes |0\rangle$, the matter-fermion eigenstate, $|\psi_{\mathbf{k}}\rangle = (\psi_{\mathbf{k}}^\dagger |\omega\rangle) \otimes |0\rangle$, the flux-pair eigenstate, $|\tilde{\chi}_{\mathbf{r}}^\alpha\rangle = |\phi_{\mathbf{r}}^\alpha\rangle \otimes [(\chi_{\mathbf{r}}^\alpha)^\dagger |0\rangle]$, and the approximate eigenstate containing both a matter fermion and a flux pair, $|\tilde{\chi}_{\mathbf{r}}^\alpha \psi_{-\mathbf{k}}\rangle = (\psi_{-\mathbf{k}}^\dagger |\phi_{\mathbf{r}}^\alpha\rangle) \otimes [(\chi_{\mathbf{r}}^\alpha)^\dagger |0\rangle]$, where $\chi_{\mathbf{r}\in A}^\alpha = \frac{1}{2}(b_{\mathbf{r}}^\alpha + i b_{\mathbf{r}+\hat{\mathbf{r}}_\alpha}^\alpha)$ are the bond fermions of the pure Kitaev model, $|0\rangle$ is the vacuum of these bond fermions, $|\omega\rangle$ is the matter-fermion ground state of the flux-free sector, and $|\phi_{\mathbf{r}}^\alpha\rangle$ is the matter-fermion ground state of the flux sector with a single flux pair around the α bond connecting the sites $\mathbf{r} \in A$ and $(\mathbf{r} + \hat{\mathbf{r}}_\alpha) \in B$. According to Eq. (S2), these matrix elements must be equal to

$$\begin{aligned}\langle \tilde{\chi}_{\mathbf{r}}^\alpha | \mathcal{H}_h | \psi_{\mathbf{k}} \rangle &= \frac{ih}{\sqrt{N}} e^{i\mathbf{k}\cdot\mathbf{r}} \left(P_{\mathbf{k},\alpha} + P_{-\mathbf{k},\alpha} e^{i\mathbf{k}\cdot\hat{\mathbf{r}}_\alpha - i\varphi_{\mathbf{k}}} \right), \\ \langle \tilde{\chi}_{\mathbf{r}}^\alpha \psi_{-\mathbf{k}} | \mathcal{H}_h | \Omega \rangle &= -\frac{ih}{\sqrt{N}} e^{i\mathbf{k}\cdot\mathbf{r}} \left(P_{\mathbf{k},\alpha} - P_{-\mathbf{k},\alpha} e^{i\mathbf{k}\cdot\hat{\mathbf{r}}_\alpha - i\varphi_{\mathbf{k}}} \right),\end{aligned} \tag{S3}$$

and, therefore, the hybridization parameters $P_{\mathbf{k},\alpha}$ are found to be

$$\begin{aligned}P_{\mathbf{k},\alpha} &= -\frac{i\sqrt{N}}{2h} e^{-i\mathbf{k}\cdot\mathbf{r}} \left[\langle \tilde{\chi}_{\mathbf{r}}^\alpha | \mathcal{H}_h | \psi_{\mathbf{k}} \rangle - \langle \tilde{\chi}_{\mathbf{r}}^\alpha \psi_{-\mathbf{k}} | \mathcal{H}_h | \Omega \rangle \right] \\ &= -\frac{i\sqrt{N}}{2} e^{-i\mathbf{k}\cdot\mathbf{r}} \left[\langle \phi_{\mathbf{r}}^\alpha | \left(i c_{\mathbf{r}} - c_{\mathbf{r}+\hat{\mathbf{r}}_\alpha} \right) \psi_{\mathbf{k}}^\dagger | \omega \rangle + \langle \phi_{\mathbf{r}}^\alpha | \psi_{-\mathbf{k}} \left(i c_{\mathbf{r}} - c_{\mathbf{r}+\hat{\mathbf{r}}_\alpha} \right) | \omega \rangle \right] \\ &= \frac{\sqrt{N}}{2} e^{-i\mathbf{k}\cdot\mathbf{r}} \left[\langle \phi_{\mathbf{r}}^\alpha | \left(c_{\mathbf{r}} + i c_{\mathbf{r}+\hat{\mathbf{r}}_\alpha} \right) \psi_{\mathbf{k}}^\dagger | \omega \rangle + \langle \phi_{\mathbf{r}}^\alpha | \left\{ \psi_{-\mathbf{k}}, c_{\mathbf{r}} + i c_{\mathbf{r}+\hat{\mathbf{r}}_\alpha} \right\} | \omega \rangle - \langle \phi_{\mathbf{r}}^\alpha | \left(c_{\mathbf{r}} + i c_{\mathbf{r}+\hat{\mathbf{r}}_\alpha} \right) \psi_{-\mathbf{k}} | \omega \rangle \right],\end{aligned} \tag{S4}$$

where $\{a, b\} \equiv ab + ba$. Setting $\mathbf{r} = \mathbf{0}$ without loss of generality, these hybridization parameters then become

$$\begin{aligned}P_{\mathbf{k},\alpha} &= \frac{\sqrt{N}}{2} \langle \phi_{\mathbf{0}}^\alpha | \left(c_{\mathbf{0}} + i c_{\hat{\mathbf{r}}_\alpha} \right) \psi_{\mathbf{k}}^\dagger | \omega \rangle + \frac{1}{2} \langle \phi_{\mathbf{0}}^\alpha | \omega \rangle \left(1 - e^{i\mathbf{k}\cdot\hat{\mathbf{r}}_\alpha - i\varphi_{\mathbf{k}}} \right) \\ &= \frac{1}{2} \sum_{\mathbf{k}'} \langle \phi_{\mathbf{0}}^\alpha | \left[\psi_{\mathbf{k}'} \left(1 + e^{i\mathbf{k}'\cdot\hat{\mathbf{r}}_\alpha - i\varphi_{\mathbf{k}'}} \right) + \psi_{\mathbf{k}'}^\dagger \left(1 - e^{-i\mathbf{k}'\cdot\hat{\mathbf{r}}_\alpha + i\varphi_{\mathbf{k}'}} \right) \right] \psi_{\mathbf{k}}^\dagger | \omega \rangle + \frac{1}{2} \langle \phi_{\mathbf{0}}^\alpha | \omega \rangle \left(1 - e^{i\mathbf{k}\cdot\hat{\mathbf{r}}_\alpha - i\varphi_{\mathbf{k}}} \right) \\ &= \langle \phi_{\mathbf{0}}^\alpha | \omega \rangle + \frac{1}{2} \sum_{\mathbf{k}'} \left(1 - e^{-i\mathbf{k}'\cdot\hat{\mathbf{r}}_\alpha + i\varphi_{\mathbf{k}'}} \right) \langle \phi_{\mathbf{0}}^\alpha | \psi_{\mathbf{k}'}^\dagger \psi_{\mathbf{k}}^\dagger | \omega \rangle.\end{aligned} \tag{S5}$$

Importantly, each matter fermion $\psi_{\mathbf{k}}$ with a given energy $\varepsilon_{\mathbf{k}}$ belongs to a degenerate set of matter fermions whose momenta $\mathbf{k}$ are related by the various symmetries of the Kitaev model. From the perspective of these matter fermions, the presence of a flux pair is a local perturbation which only affects the two sites $\mathbf{0} \in A$ and $\hat{\mathbf{r}}_\alpha \in B$ connected by the corresponding α bond. Therefore, we can form appropriate linear combinations of the degenerate matter fermions such that only two linear combinations couple to

the perturbation while the remaining ones have vanishing wave functions at both sites $\mathbf{0}$ and $\hat{\mathbf{r}}_\alpha$. Exploiting the residual inversion symmetry around the flux pair (i.e., the corresponding α bond), the natural choice for these two linear combinations is

$$\psi_{\varepsilon,\pm} = \mathcal{N}_{\varepsilon,\pm}^{-1} \sum_{\{\mathbf{k}\}_\varepsilon} \left(1 \pm e^{i\mathbf{k}\cdot\hat{\mathbf{r}}_\alpha - i\varphi_\mathbf{k}}\right) \psi_\mathbf{k}, \qquad \mathcal{N}_{\varepsilon,\pm} = \sqrt{\sum_{\{\mathbf{k}\}_\varepsilon} \left|1 \pm e^{i\mathbf{k}\cdot\hat{\mathbf{r}}_\alpha - i\varphi_\mathbf{k}}\right|^2}, \tag{S6}$$

where $\{\mathbf{k}\}_\varepsilon$ is the set of all momenta $\mathbf{k}$ satisfying $\varepsilon_\mathbf{k} = \varepsilon$. Since the matter fermions $\psi_{\varepsilon,\pm}$ have eigenvalues $\pm i$ under the residual inversion symmetry (which acts projectively on the matter fermions), the two-fermion matrix element in Eq. (S5) then becomes

$$\langle \phi_\mathbf{0}^\alpha | \psi_{\mathbf{k}'}^\dagger \psi_\mathbf{k}^\dagger | \omega \rangle = \sum_\pm \mathcal{N}_{\varepsilon_{\mathbf{k}'},\pm}^{-1} \mathcal{N}_{\varepsilon_\mathbf{k},\mp}^{-1} \left(1 \pm e^{i\mathbf{k}'\cdot\hat{\mathbf{r}}_\alpha - i\varphi_{\mathbf{k}'}}\right) \left(1 \mp e^{i\mathbf{k}\cdot\hat{\mathbf{r}}_\alpha - i\varphi_\mathbf{k}}\right) \langle \phi_\mathbf{0}^\alpha | \psi_{\varepsilon_{\mathbf{k}'},\pm}^\dagger \psi_{\varepsilon_\mathbf{k},\mp}^\dagger | \omega \rangle. \tag{S7}$$

Note that $\langle \phi_\mathbf{0}^\alpha | \psi_{\varepsilon_{\mathbf{k}'},+}^\dagger \psi_{\varepsilon_\mathbf{k},+}^\dagger | \omega \rangle = \langle \phi_\mathbf{0}^\alpha | \psi_{\varepsilon_{\mathbf{k}'},-}^\dagger \psi_{\varepsilon_\mathbf{k},-}^\dagger | \omega \rangle = 0$ because of inversion symmetry. Using the auxiliary identities

$$\begin{aligned} \sum_{\{\mathbf{k}\}_\varepsilon} \left(1 - e^{i\mathbf{k}\cdot\hat{\mathbf{r}}_\alpha - i\varphi_\mathbf{k}}\right) \left(1 - e^{-i\mathbf{k}\cdot\hat{\mathbf{r}}_\alpha + i\varphi_\mathbf{k}}\right) &= \mathcal{N}_{\varepsilon,-}^2, \\ \sum_{\{\mathbf{k}\}_\varepsilon} \left(1 + e^{i\mathbf{k}\cdot\hat{\mathbf{r}}_\alpha - i\varphi_\mathbf{k}}\right) \left(1 - e^{-i\mathbf{k}\cdot\hat{\mathbf{r}}_\alpha + i\varphi_\mathbf{k}}\right) &= 0, \end{aligned} \tag{S8}$$

the momentum-space hybridization parameters in Eq. (S5) finally take the form

$$P_{\mathbf{k},\alpha} = \langle \phi_\mathbf{0}^\alpha | \omega \rangle + \frac{1}{2} \left(1 + e^{i\mathbf{k}\cdot\hat{\mathbf{r}}_\alpha - i\varphi_\mathbf{k}}\right) \mathcal{N}_{\varepsilon_\mathbf{k},+}^{-1} \sum_{\varepsilon'} \mathcal{N}_{\varepsilon',-} \langle \phi_\mathbf{0}^\alpha | \psi_{\varepsilon',-}^\dagger \psi_{\varepsilon_\mathbf{k},+}^\dagger | \omega \rangle \equiv C + A(\varepsilon_\mathbf{k}) \left[1 + e^{i\mathbf{k}\cdot\hat{\mathbf{r}}_\alpha - i\varphi_\mathbf{k}}\right]. \tag{S9}$$

It can be shown numerically (and argued analytically) that, at the lowest energies, $\varepsilon' \to 0$ and $\varepsilon_\mathbf{k} \to 0$, the leading-order behavior of the two-fermion matrix element in Eq. (S9) is given by $\langle \phi_\mathbf{0}^\alpha | \psi_{\varepsilon',-}^\dagger \psi_{\varepsilon_\mathbf{k},+}^\dagger | \omega \rangle \sim (\varepsilon' + \varepsilon_\mathbf{k})^{-1}$. Therefore, the schematic form of $A(\varepsilon_\mathbf{k})$ around $\varepsilon_\mathbf{k} = 0$, corresponding to the K point of the Brillouin zone, becomes $A(\varepsilon_\mathbf{k}) \sim \int_0^\Lambda d\varepsilon' g(\varepsilon') [\varepsilon' + \varepsilon_\mathbf{k}]^{-1} \sim A_0 + A_1 \varepsilon_\mathbf{k} \ln \varepsilon_\mathbf{k}$, where $g(\varepsilon) \sim \varepsilon$ is the low-energy matter-fermion density of states. Consequently, while $A(\varepsilon_\mathbf{k})$ is finite at $\varepsilon_\mathbf{k} = 0$, it is not analytic because its derivative diverges. Moreover, the factor $e^{-i\varphi_\mathbf{k}}$ has a nontrivial phase winding around the K point. This nonanalytic behavior of the hybridization function $P_{\mathbf{k},\alpha}$ reflects the gapless Dirac cone of the matter fermions. However, the matter fermions are known to be gapped out by an infinitesimally small magnetic field. Thus, a finite field should remove the nonanalytic behavior by generating an exponential decay for the real-space hybridization function $p_{\mathbf{R},\alpha}$. On a phenomenological level, we can account for this exponential decay by multiplying $p_{\mathbf{R},\alpha}$ with $\exp(-|\mathbf{R}|/\xi)$, which is equivalent to taking a convolution between $P_{\mathbf{k},\alpha}$ and a Lorentzian function of width $\sim 1/\xi$ in momentum space. We emphasize that, while this regularization procedure is important for producing the correct field dependence of the gap opening at the K point, it has negligible effects on all the other results of this work. In practice, we take $\xi = 25$ for the hybridization decay length.

To determine the hopping parameter q, we match the flux-pair hopping term in Eq. (3) of the main text with the microscopic model by considering the matrix element of the bare Zeeman term, $\mathcal{H}_h = h \sum_\alpha \sum_\mathbf{r} \sigma_\mathbf{r}^\alpha$, where $\sigma_\mathbf{r}^\alpha = i b_\mathbf{r}^\alpha c_\mathbf{r} = -\frac{i}{2} \sum_{\beta,\gamma} \epsilon_{\alpha\beta\gamma} b_\mathbf{r}^\beta b_\mathbf{r}^\gamma$, between the flux-pair eigenstates $|\tilde{\chi}_\mathbf{0}^x\rangle = |\phi_\mathbf{0}^x\rangle \otimes [(\chi_\mathbf{0}^x)^\dagger |0\rangle]$ and $|\tilde{\chi}_\mathbf{0}^y\rangle = |\phi_\mathbf{0}^y\rangle \otimes [(\chi_\mathbf{0}^y)^\dagger |0\rangle]$ of the pure Kitaev model. The hopping parameter is then found to be

$$q = \frac{i}{h} \langle \tilde{\chi}_\mathbf{0}^x | \mathcal{H}_h | \tilde{\chi}_\mathbf{0}^y \rangle = i \langle \tilde{\chi}_\mathbf{0}^x | \sigma_\mathbf{0}^z | \tilde{\chi}_\mathbf{0}^y \rangle + i \langle \tilde{\chi}_\mathbf{0}^x | \sigma_{\hat{\mathbf{r}}_z}^z | \tilde{\chi}_\mathbf{0}^y \rangle = \langle \phi_\mathbf{0}^x | \phi_\mathbf{0}^y \rangle + \langle \phi_\mathbf{0}^x | i c_\mathbf{0} c_{\hat{\mathbf{r}}_z} | \phi_\mathbf{0}^y \rangle \equiv q_0 + q'. \tag{S10}$$

Also, we can straightforwardly determine the signs of q_0 and q' by considering appropriate products of the corresponding matrix elements $\langle \tilde{\chi}_\mathbf{0}^x | \sigma_\mathbf{0}^z | \tilde{\chi}_\mathbf{0}^y \rangle = -i q_0$ and $\langle \tilde{\chi}_\mathbf{0}^x | \sigma_{\hat{\mathbf{r}}_z}^z | \tilde{\chi}_\mathbf{0}^y \rangle = -i q'$ (along with their cyclic permutations in x, y, z):

$$\begin{aligned} P_1 &= \langle \tilde{\chi}_\mathbf{0}^y | \sigma_\mathbf{0}^x | \tilde{\chi}_\mathbf{0}^z \rangle \langle \tilde{\chi}_\mathbf{0}^z | \sigma_\mathbf{0}^y | \tilde{\chi}_\mathbf{0}^x \rangle \langle \tilde{\chi}_\mathbf{0}^x | \sigma_\mathbf{0}^z | \tilde{\chi}_\mathbf{0}^y \rangle = i q_0^3, \\ P_2 &= \langle \tilde{\chi}_\mathbf{0}^y | \sigma_\mathbf{0}^z | \tilde{\chi}_\mathbf{0}^x \rangle \langle \tilde{\chi}_\mathbf{0}^x | \sigma_{\hat{\mathbf{r}}_z}^z | \tilde{\chi}_\mathbf{0}^y \rangle = q_0 q'. \end{aligned} \tag{S11}$$

Since the individual matrix elements are expected to be $O(1)$ due to the absence of an orthogonality catastrophe, it is a reasonable approximation to neglect the projectors to the intermediate states. In this approximation, the products in Eq. (S11) become

$$\begin{aligned} P_1 &\approx \langle \tilde{\chi}_\mathbf{0}^y | \sigma_\mathbf{0}^x \sigma_\mathbf{0}^y \sigma_\mathbf{0}^z | \tilde{\chi}_\mathbf{0}^y \rangle = i, \\ P_2 &\approx \langle \tilde{\chi}_\mathbf{0}^y | \sigma_\mathbf{0}^z \sigma_{\hat{\mathbf{r}}_z}^z | \tilde{\chi}_\mathbf{0}^y \rangle = \langle \sigma_\mathbf{0}^z \sigma_{\hat{\mathbf{r}}_z}^z \rangle < 0. \end{aligned} \tag{S12}$$

From a comparison between Eqs. (S11) and (S12), we conclude that q_0 is positive while q' is negative.

II. EXPECTATION VALUE OF THE FLUX OPERATOR

Here we describe how the expectation value of the $\mathbb{Z}_2$ gauge-flux operator W_p in Eq. (2) of the main text can be computed for the effective Hamiltonian $\tilde{\mathcal{H}}$ in Eq. (3) in the main text. We first recognize that the dressed bond-fermion operators $\tilde{\chi}_{\mathbf{r}}^{\alpha}$ and the bare bond-fermion operators $\chi_{\mathbf{r}}^{\alpha}$ have exactly the same effect on the flux degrees of freedom as they only differ in an appropriate distortion of the matter-fermion state. Therefore, in terms of the dressed bond variables $\tilde{u}_{jj'}^{\alpha} = i\tilde{b}_j^{\alpha}\tilde{b}_{j'}^{\alpha}$ and the ground state $|\tilde{\Omega}\rangle$ of the quadratic fermion Hamiltonian $\tilde{\mathcal{H}}$, the expectation value of the flux operator W_p becomes

$$\langle W_p \rangle = \langle \tilde{\Omega} | \tilde{u}_{12}^{z} \tilde{u}_{32}^{x} \tilde{u}_{34}^{y} \tilde{u}_{54}^{z} \tilde{u}_{56}^{x} \tilde{u}_{16}^{y} | \tilde{\Omega} \rangle = -\langle \tilde{\Omega} | (\tilde{b}_1^z \tilde{b}_2^z)(\tilde{b}_3^x \tilde{b}_2^x)(\tilde{b}_3^y \tilde{b}_4^y)(\tilde{b}_5^z \tilde{b}_4^z)(\tilde{b}_5^x \tilde{b}_6^x)(\tilde{b}_1^y \tilde{b}_6^y) | \tilde{\Omega} \rangle, \tag{S13}$$

where the subscript $j = 1, 2, \ldots, 6$ labels the six sites around the hexagon p (see Fig. 1 of the main text). In turn, this expectation value can be computed by means of Wick's theorem, which reduces the 12-fermion expectation value to products of two-fermion expectation values, $\langle \tilde{b}_{\boldsymbol{r}}^{\alpha} \tilde{b}_{\boldsymbol{r}'}^{\alpha'} \rangle = \langle \tilde{\Omega} | \tilde{b}_{\boldsymbol{r}}^{\alpha} \tilde{b}_{\boldsymbol{r}'}^{\alpha'} | \tilde{\Omega} \rangle$. If we then write the bond-fermion operators $\tilde{b}_{\boldsymbol{r}}^{\alpha}$ in terms of the fermion eigenmodes $\gamma_{n,\boldsymbol{k}}$ (with $n = 1, 2, \ldots, 8$) of the effective Hamiltonian,

$$\tilde{b}_{\boldsymbol{r}\in\nu}^{\alpha} = \frac{1}{\sqrt{N}} \sum_{n,\boldsymbol{k}} \left[(u_{n,-\boldsymbol{k}}^{\alpha,\nu})^* \gamma_{n,\boldsymbol{k}} + u_{n,\boldsymbol{k}}^{\alpha,\nu} \gamma_{n,-\boldsymbol{k}}^{\dagger} \right] e^{i\boldsymbol{k}\cdot\boldsymbol{r}}, \qquad \nu = A, B, \tag{S14}$$

where $u_{n,\boldsymbol{k}}^{\alpha,\nu}$ are obtained from a straightforward diagonalization of $\tilde{\mathcal{H}}$, each two-fermion expectation value takes the form

$$\langle \tilde{b}_{\boldsymbol{r}\in\nu}^{\alpha} \tilde{b}_{\boldsymbol{r}'\in\nu'}^{\alpha'} \rangle = \frac{1}{N} \sum_{n,\boldsymbol{k}} (u_{n,\boldsymbol{k}}^{\alpha,\nu})^* u_{n,\boldsymbol{k}}^{\alpha',\nu'} e^{i\boldsymbol{k}\cdot(\boldsymbol{r}'-\boldsymbol{r})}. \tag{S15}$$

While the ground state $|\tilde{\Omega}\rangle$ contains no bond fermions for $h = 0$, corresponding to $\langle W_p \rangle = 1$, the hybridization between the bond fermions and the matter fermions leads to a finite density of bond fermions for $h > 0$, which corresponds to $\langle W_p \rangle < 1$.

III. COEFFICIENTS OF THE EFFECTIVE FIELD THEORY

Here we provide the coefficients of the effective field theory in Eq. (11) of the main text. These coefficients can be computed by projecting the effective Hamiltonian in Eq. (4) of the main text to the two low-energy fermion bands corresponding to Eq. (9) of the main text. For concreteness, the momentum $\boldsymbol{k} \equiv (k_x, k_y)$ is described in Cartesian coordinates defined by the unit vectors $\mathbf{e}_x \parallel \hat{\mathbf{r}}_y - \hat{\mathbf{r}}_x$ and $\mathbf{e}_y \parallel \hat{\mathbf{r}}_z$ [see Fig. 1 of the main text for definitions of $\hat{\mathbf{r}}_\alpha$], while the length unit is taken as the lattice constant a (i.e., the distance between two neighboring A sites).

From a long-wavelength expansion around the Γ point, we obtain the following analytical expressions:

$$\begin{aligned}
c_0' &= -\frac{\Delta_\chi}{6+\Delta_\chi} \frac{12}{h_c}, \\
c_z &= \frac{\Delta_\chi}{6+\Delta_\chi} \left[3\left(\frac{C}{U}\right)^2 \frac{1}{1-(h_c/h_c')^2} - \frac{3}{4} - \frac{12V}{U} \right], \\
c_{xx} &= -\frac{\Delta_\chi}{2(6+\Delta_\chi)} \frac{C}{U} \frac{h_c/h_c'}{1-(h_c/h_c')^2}, \quad c_{yy} = \frac{h_c}{h_c'} c_{xx}, \\
c_{xy} &= c_{yx} = 0,
\end{aligned}$$

where $h_c = \sqrt{6\Delta_\chi}/U$ and $h_c' = \Delta_\chi/(2\sqrt{3}q)$ are the two critical fields corresponding to the Γ point [see the main text], $C = \langle \phi_{\mathbf{0}}^{\alpha} | \omega \rangle$ [see Eq. (S9)], while U and V are defined by $(2/\sqrt{3}) \sum_\alpha P_{\mathbf{k},\alpha} = U + Vk^2 + O(k^3)$. For a finite honeycomb lattice with $N = 121 \times 121$ unit cells, we numerically obtain $C/U \simeq 0.3507$, $h_c/h_c' \simeq 0.3284$, and $V/U \simeq -0.0532$. Therefore, the coefficients in Eq. (**??**) are found to be $c_0' \simeq -1.00$, $c_z \simeq 0.0125$, $c_{xx} \simeq -0.00268$, and $c_{yy} \simeq -0.00088$.

IV. NONANALYTIC BEHAVIOR OF THE GROUND-STATE ENERGY

Here we analyze the nonanalytic behavior of the ground-state energy $E_G(h)$ at the critical field $h = h_c$. Specifically, we show that the second derivative, $E_G'' = d^2 E_G / dh^2$, is discontinuous at $h = h_c$, and provide an expression for its discontinuity, $\Delta E_G''$, in terms of the effective field theory [see Eqs. (8) and (11) of the main text]. Given the infrared nature of the singularity, it is useful

to write the ground-state energy as a sum of two contributions, $E_G = \tilde{E}_G(\Lambda) + \delta E_G(\Lambda)$, which correspond to the long-wavelength modes with momentum $k \equiv |\mathbf{k}| < \Lambda$ and the remaining modes with momentum $k > \Lambda$, respectively:

$$\tilde{E}_G(\Lambda) = -\int_{k<\Lambda} \frac{d^2\mathbf{k}}{\Omega} \frac{\omega_{\mathbf{k}}}{2}, \quad \delta E_G(\Lambda) = -\int_{\mathrm{BZ},k>\Lambda} \frac{d^2\mathbf{k}}{\Omega} \frac{\omega_{\mathbf{k}}}{2}, \tag{S16}$$

where $\Omega = 8\pi^2/\sqrt{3}$ is the area of the Brillouin zone (in units of a^{-2}), $\Lambda \ll 1$ is an arbitrary cutoff, and $\omega_{\mathbf{k}}$ is the energy of the mode at momentum $\mathbf{k}$ [see Eq. (12) of the main text]. The second contribution $\delta E_G(\Lambda)$ is analytic at $h = h_c$ because all of its derivatives are well defined. Therefore,

$$\Delta E_G'' = \lim_{h\to h_c^-} E_G'' - \lim_{h\to h_c^+} E_G'' = \lim_{\Lambda\to 0} \left[\lim_{h\to h_c^-} \tilde{E}_G''(\Lambda) - \lim_{h\to h_c^+} \tilde{E}_G''(\Lambda) \right]. \tag{S17}$$

In other words, the discontinuity in E_G'' at the critical field can be completely extracted from the first contribution in the $\Lambda \to 0$ limit (i.e., the effective field theory) because it does not depend on the cutoff Λ.

From Eq. (12) of the main text, the second derivative of $\tilde{E}_G(\Lambda)$ with respect to the field h is given by

$$\tilde{E}_G''(\Lambda) = -\int_{k<\Lambda} \frac{d^2\mathbf{k}}{\Omega} \left\{ \frac{1}{2\omega_{\mathbf{k}}} \sum_{\mu=x,y,z} \left[\left(\frac{d\beta_{\mathbf{k}}^{\mu}}{dh} \right)^2 + \beta_{\mathbf{k}}^{\mu} \frac{d^2\beta_{\mathbf{k}}^{\mu}}{dh^2} \right] - \frac{1}{2\omega_{\mathbf{k}}^3} \left(\sum_{\mu=x,y,z} \beta_{\mathbf{k}}^{\mu} \frac{d\beta_{\mathbf{k}}^{\mu}}{dh} \right)^2 \right\}. \tag{S18}$$

In general, the functions $\beta_{\mathbf{k}}^{\mu}$ depend on the field h via the coefficients c_0, c_z, and $c_{\eta\nu}$ in Eq. (11) of the main text. However, in the $\Lambda \to 0$ limit, the field derivatives of c_z and $c_{\eta\nu}$ are necessarily subdominant with respect to the field derivatives of c_0 as they are multiplied by small factors $k^2 < \Lambda^2$ and $k^3 < \Lambda^3$, respectively. Therefore, we focus exclusively on the field dependence of the function $\beta_{\mathbf{k}}^{z}$ via the coefficient c_0. If we expand this coefficient around $h = h_c$ as $c_0(h) = c_0'(h-h_c) + \frac{1}{2}c_0''(h-h_c)^2 + O(h-h_c)^3$, the second derivative of $\tilde{E}_G(\Lambda)$ close to the critical field ($h \simeq h_c$) becomes

$$\tilde{E}_G''(\Lambda) = -\int_{k<\Lambda} \frac{d^2\mathbf{k}}{\Omega} \frac{(\beta_{\mathbf{k}}^{x})^2 + (\beta_{\mathbf{k}}^{y})^2}{2\omega_{\mathbf{k}}^3} (c_0')^2 - \int_{k<\Lambda} \frac{d^2\mathbf{k}}{\Omega} \frac{\beta_{\mathbf{k}}^{z}}{2\omega_{\mathbf{k}}} c_0'' \equiv \left[\tilde{E}_G''(\Lambda)\right]^{(1)} + \left[\tilde{E}_G''(\Lambda)\right]^{(2)}. \tag{S19}$$

We first recognize that the second integral in Eq. (S19) vanishes in the $\Lambda \to 0$ limit for both $h \to h_c^+$ and $h \to h_c^-$. Indeed, since $|\beta_{\mathbf{k}}^{z}/\omega_{\mathbf{k}}| \leq 1$, the magnitude of this integral has an $O(\Lambda^2)$ upper bound:

$$\left| \left[\tilde{E}_G''(\Lambda)\right]^{(2)} \right| = \left| \int_{k<\Lambda} \frac{d^2\mathbf{k}}{\Omega} \frac{\beta_{\mathbf{k}}^{z}}{2\omega_{\mathbf{k}}} c_0'' \right| \leq \int_{k<\Lambda} \frac{d^2\mathbf{k}}{\Omega} \frac{|c_0''|}{2} = \frac{\sqrt{3}\Lambda^2 |c_0''|}{16\pi}. \tag{S20}$$

In contrast, the first integral in Eq. (S19) has completely different behaviors for $h \to h_c^+$ and $h \to h_c^-$. To analyze this integral, it is helpful to use polar coordinates, $\mathbf{k} = (k\cos\theta, k\sin\theta)$, write $\beta_{\mathbf{k}}^{\eta=x,y} = k^3 \sum_{\nu=x,y} c_{\eta\nu} f_\nu(\theta)$ in terms of $f_x(\theta) = \cos\theta(3\sin^2\theta - \cos^2\theta)$ and $f_y(\theta) = \sin\theta(3\cos^2\theta - \sin^2\theta)$, and introduce the positive-definite angular function

$$F(\theta) = k^{-6} \left[(\beta_{\mathbf{k}}^{x})^2 + (\beta_{\mathbf{k}}^{y})^2 \right] = \sum_{\eta,\nu=x,y} \left[c_{\eta\nu} f_\nu(\theta) \right]^2. \tag{S21}$$

Using $\beta_{\mathbf{k}}^{z} \simeq |c_0'|(h_c - h) + c_z k^2$ (where $c_0' < 0$ and $c_z > 0$), the first integral in Eq. (S19) can then be written as

$$\left[\tilde{E}_G''(\Lambda)\right]^{(1)} = -\frac{\sqrt{3}}{4} \left(\frac{c_0'}{2\pi} \right)^2 \int_0^{2\pi} d\theta \int_0^{\Lambda} dk \, \frac{k^7 F(\theta)}{\left\{ k^6 F(\theta) + \left[|c_0'|(h_c - h) + c_z k^2 \right]^2 \right\}^{3/2}}. \tag{S22}$$

Below the critical field, $h < h_c$, the denominator of the integrand is bounded from below by $c_z^3 k^6$. Thus, the integral vanishes in the $\Lambda \to 0$ limit for $h \to h_c^-$ because its magnitude has an $O(\Lambda^2)$ upper bound:

$$\left| \lim_{h\to h_c^-} \left[\tilde{E}_G''(\Lambda)\right]^{(1)} \right| \leq \frac{\sqrt{3}}{4} \left(\frac{c_0'}{2\pi} \right)^2 \int_0^{2\pi} d\theta \int_0^{\Lambda} dk \, \frac{k F_{\max}}{c_z^3} = \frac{\sqrt{3}\Lambda^2 (c_0')^2 F_{\max}}{16\pi c_z^3}, \tag{S23}$$

where $F_{\max} = \max_\theta F(\theta)$. In contrast, above the critical field, $h > h_c$, the denominator of the integrand in Eq. (S22) is very small along the low-energy ring of radius $K = \sqrt{|c_0'|(h - h_c)/c_z}$. Expanding the integrand around this radius K by introducing a new radial variable, $x = (k - K)/K^2$, the integral in Eq. (S22) then becomes

$$\left[\tilde{E}_G''(\Lambda)\right]^{(1)} = -\frac{\sqrt{3}}{4} \left(\frac{c_0'}{2\pi} \right)^2 \int_0^{2\pi} d\theta \int_{-1/K}^{(\Lambda-K)/K^2} dx \, \frac{K^9 (1+Kx)^7 F(\theta)}{\left\{ K^6 (1+Kx)^6 F(\theta) + \left[-c_z K^2 + c_z K^2 (1+Kx)^2 \right]^2 \right\}^{3/2}}. \tag{S24}$$

Next, in the limit of $h \longrightarrow h_c^+$, corresponding to $K \longrightarrow 0$, the integral reduces to

$$\lim_{h\to h_c^+} \left[\tilde{E}_G''(\Lambda)\right]^{(1)} = -\frac{\sqrt{3}}{4}\left(\frac{c_0'}{2\pi}\right)^2 \int_0^{2\pi} d\theta \int_{-\infty}^{+\infty} dx \, \frac{F(\theta)}{\left[F(\theta)+4c_z^2x^2\right]^{3/2}} = -\frac{\sqrt{3}\,(c_0')^2}{8\pi c_z}. \tag{S25}$$

This K-independent result has a simple interpretation: the first integral in Eq. (S19) is dominated by an annulus of radius K and width $O(K^2)$ in which the integrand is $O(K^{-3})$. Finally, by collecting the results from Eqs. (S20), (S23), and (S25), we conclude that the discontinuity in the second derivative of the ground-state energy at the critical field is given by

$$\Delta E_G'' = \lim_{h\to h_c^-} E_G'' - \lim_{h\to h_c^+} E_G'' = \lim_{\Lambda\to 0}\left[\lim_{h\to h_c^-} \tilde{E}_G''(\Lambda) - \lim_{h\to h_c^+} \tilde{E}_G''(\Lambda)\right] = \frac{\sqrt{3}\,(c_0')^2}{8\pi c_z}. \tag{S26}$$

Remarkably, the discontinuity only depends on two parameters, c_0' and c_z, of the effective field theory.
